# On the Teeth as an Indication of the Progressive Improvement of the Human Race

**Published:** 1851-07

**Authors:** 


### ARTICLE XX.

### *On the Teeth as an Indication of the Progressive Improvement of the Human Race.*

There is another important view in which the anatomy of these parts, in their progressive development, may be considered; one which bears intimately on the subject of ethnology, and which, in consequence, I took the opportunity of laying before the Ethnological Society of London in the following communication.*

* On the human mouth, read April 23, 1845.

"Glancing at the different degrees of development in man which come within notice, and the various features found to be prevalent, and made use of, with a view to characterize the varieties of man, we find them to be numerous, and to produce much diversity of appearance.

"When we observe the difference between the European and the negro in color; the long, flowing, light-colored hair of the Caucasian, and the black woolly hair of the negro; the well-balanced, elevated, and finely-symmetrical cranium of the Caucasian; the extremely prominent and well-furnished mouth of the negro, and the pinched perpendicular mouth, supplied with irregularly-arranged and imperfectly-organized teeth of common life—the question may well be asked—Has man descended from a state of perfection or risen from a low and deficient condition of development?

"The arguments which have been advanced on this subject have generally tended towards the adoption of one or other extreme in the scale of development, with a view to solve the difficulties regarding the original stock whence mankind have sprung. Here we must exclusively take into consideration possibilities, and these so far only as they are consistent with the experience and evidence of facts within our reach. We have to contemplate the natural scene of existence into which man must originally have been ushered. The development compatible with the due fulfilment of the exactions required from such a being, in such a state of existence, must, in my opinion, have been perfect, and one well-balanced both in its moral and physical attributes. A mind of morbid sensibility, such as highly cultivated social life in all ages presents, would have sunk under the exactions inevitable to such a state. It would not have been able to exert the requisite force to combat them, and it would have been too sensitive to have allowed man to have acted as a creature of simple instinct.

"If, on the other hand, the development of his physical frame and moral attributes had been of a low standard, he would neither have been possessed of strength and vigor adequate to contend with the peculiarities of his state of existence, nor have had mind to comprehend, nor judgment to regulate it. He would have been totally defenceless against the violence of the elements, and the attacks of the animated creatures around him—man being naturally defenceless, and deriving all his power from the regulation and direction of his rational faculties. If the origin of mankind had really been that of a low and degraded scale of development, even if such a state were compatible with his existence, it does not seem to me that emancipation from it could have been possible. I am, therefore, at issue with Dr. Pritchard in the opinion expressed by him, that it must be concluded that the process of nature in the human race is the transmutation of the characters of the negro into those of the European. Such a view is not the result of my researches.

"I hope to show that there is no difficulty in supposing a derivation from one original stock, and that certainly the origin of the varieties in the development of the mouth must have been from a perfect type. The capability of the existence of man in different climates is only bounded by the extreme limits of the globe; his assimilative functions are omnivorous; his powers of articulation unlimited; and his physical capabilities combine all the possible modifications of the lower animals whose spheres of action are terrestrial. His mental powers are of the highest order; and, when we see that the inferior animals are endowed to so great an extent with plasticity and power of accommodation to circumstances, surely we cannot possibly deny to man a power of individual and hereditary adaptation adequate to fit him for the perfect enjoyment of such versatility.

"In regard to the form of the head, which presents the most notable ethnological marks, various points have been attended to—in fact, the relative proportions of every salient part. In reviewing the observations which have been made thereon, so far as they are connected with ethnology, no feature seems to me to bear so instructively on the solution of the various difficult problems involved in this study as the form of the mouth and the development of the teeth. Everything would appear to yield to the necessities of existence, and the varied materials for sustaining that existence, the manner of procuring these materials, and their situation and nature. The mouth is the original and essential constituent of the apparatus for the assimilation of these materials, and in the lower animals it is peculiarly and beautifully adapted to their exigencies. In the mouths of men, too, we observe a medium type fitted to every peculiarity of terrestrial existence, and capable of performing every office exacted from the mouth in all the lower animals. Just as those peculiarities are exacted by external circumstances and situation, so we have a display of corresponding peculiarities of organization. As I have said on another occasion, it is a remarkable fact, that no other conformation of mouth than that of man, could admit at once of perfect articulation and mastication of his varied food. This organ may be regarded as fulfilling a most essential part in his intellectual life; for it is not only in him, in common with all other animals, the essential and original element of the apparatus of assimilation, but it is also the organ of intellectual expression, and, as such, is equally indispensable to the existence of the race, and an essential agent for the improvement of man's condition, and for his communion in social life. From mere observation, therefore, of the conformity of development of the anterior chambers of the head, with the presentation of the anterior portion of the mouth, we may be led to the general conclusion, that those of weak intellect were forced originally to emigrate to the more inhospitable quarters of the globe, for we find that the inhabitants of these climates are generally possessed of a low development of forehead, with a protruded jaw; while those still inhabiting the position of the original stock possess an elevated forehead and a perpendicular jaw.

"Blumenbach raised the maxilla into a place of importance by taking his characteristic diameters of the cranium from the conjoint form of the frontal and maxillary bones; and he regards them as the most important points on which the general character of the head depends. The facial angle of Camper is a subject which still retains much interest, though that interest might probably have passed away had it not comprehended within its range the comparative development of the anterior or intellectual portion of the brain. Still, the interesting features of that portion of the subtending lines of the angle connected with the mouth, although not neglected, yet, in my opinion, require more consideration than has hitherto been allotted to them. That acquiescence in the harmony of nature, which seems to be irresistible, might probably call forth an assent to the accuracy of these general remarks; but, however close the reasoning on hypothetical principles may be, science demands demonstration from facts before we can freely or fully yield our assent to any proposition. We must inquire if deviations in the character of the mouth are simply the effect of deviations in the habits of individuals composing races; whether they are partial, and appear in individuals only, or general, and amount to a national or tribe characteristic. We know that the osseous portion of the animal frame is modelled by the soft parts, and that, in fact, the bones may be considered as mere passive accessories, affording points of attachment as well as protection to the soft parts, which are the springs by which the animal machine is worked in all its complicated movements. That passive character, however, offers, in its nature, a direct demonstration of the amount of activity of the soft parts connected with such portion of individual structure. In the present case it must be evident, and the instruction derived from the development of these parts must be regarded as direct. We must seek for the origin of the characteristic differences amongst the various groups of mankind, in causes which are natural, general, and indispensable to the existence of man in his particular position. We must also look for the origin of certain appearances in manners and customs. The form of the mouth, and the condition of the teeth, must be studied, in reference to the habits of infancy, as regulating the development, particularly as to the kind of food consumed, and weight must also be given to the effects of hereditary transference of character.

"The relative perfection of development of the organs generally, and the teeth especially, is affected by other causes, viz. the circumstances connected with general development, such as the periods of womanhood and marriage, and the habits of life, particularly of females. The nature of the food will always materially regulate the state of the teeth throughout life.

"There is a practical fact of fundamental importance, in reference to this inquiry, which will materially explain and illustrate the points under consideration. It is this, that the natural action of the lower jaw upon the upper may push out, evert, or expand the arch of the latter; but, on the other hand, it is impossible by any habitual or natural act performed by the mouth, or by the individual in any way, to bring in, or to contract that arch, so as to produce, out of the prominent jaw of the negro, the vertical or perpendicular jaw of the Caucasian. The prominent character may, therefore, be derived from the vertical, but the vertical never can be produced out of the prominent, by any habit or exercise of the function of the organ.

"The causes which produce the prominent development are palpably of common occurrence, and matters of every-day observation; and this feature of a race can only be reclaimed by the ameliorating influences of successive generations, in abstinence from practices which give rise to the eversion. Unless, indeed, the perpendicular mouth had been the original presentation of mankind, there is no exercise in which these organs could be employed, so as to develop such a feature; but I hope presently to show that the constitution of the parts individually, and of man and his manners generally, all conspire to the production of the prominent mouth from the vertical type.

"The vertical mouth is said to be the original development of the infant negro; the advanced mouth of the adult negro, therefore, is not congenital but factitious. We are also told, that the progeny of the negro of the southern provinces of the United States, owing to the different circumstances in which it is placed, has not the advanced mouth and its concomitant features after the second or third generations. It will be necessary, however, to show that these parts, are of such a plastic nature as to admit of this factitious development. Their habits and exactions will also require to be considered, for the purpose of ascertaining how they become plastic, and are factitiously modeled out of their congenital arrangement; and, with a view to understand the nature and extent of the plasticity of the osseous portion of the organ, I shall now describe the anatomy of the mouth, and show how far these parts are under the influence of the moulding and controlling powers of the muscles, in the performance of the functions required of them. As I do not, however, intend to give here a strictly anatomical demonstration, nor yet a physiological disquisition, what I shall say will consist more of a general explanation of that which is necessary to be attended to, with a view to understand my theory, than anything else. I have already alluded to the complicated nature of the operations exacted of the mouth in articulation and mastication.

"The degree of perfection in development of all the different portions of the mouth, must regulate the degree of perfection with which the work to be performed by it is accomplished. Perfectly distinct articulation is not compatible with the prominent jaw of the uncivilized, neither is it compatible with the irregularly developed mouth of the civilized; nor is it possible for the diversified exercise of the organ in the different actions exacted for the division, comminution, and grinding of food to be well performed by such mouths, as social life is every day furnishing an endless variety. The irregularity of the teeth in such mouths causes the one jaw to become locked within the other, and thereby prevents such latitude of action as is adequate to the due performance of these varied duties. Mastication is performed by means of one portion of the mouth being passive, and the other active; the under jaw, consisting of bone and muscle, is the active, the upper jaw the passive portion; but although, collectively, the under jaw is active, yet this is again resolvable into a single portion acted upon, namely, the solid bone, and a number of parts producing the action, or in which the power resides, namely, the muscles. The force employed is that of a lever of the third order, the principal force being exerted by the powerful temporal muscles inserted into the coronoid processes, and situated between the fulcrum, residing in the condyles, and the weight to be overcome produced by the substances for comminution, placed between the teeth, in any situation around the dental arch, but always anterior to the power exerted by the temporal muscles. Other muscles (the masseter and pterygoid) inserted into the under jaw, and deriving their origin from points of the bones forming the superior portion of the face, may be looked upon more in the light of controlling powers than otherwise. At the same time, they afford a certain direct assistance in elevating the under jaw, but their characteristic sphere of action is in varying and regulating the chief power produced by the temporal muscles. The principal duty of the remaining large muscle of the under jaw, the buccinator, is that of perfecting the parietes of the mouth. It forms an antagonist to the tongue in receiving the food into the mouth for mastication, and in retaining it within the influence of the grinding apparatus. This beautiful piece of machinery, taken as a whole, may be considered in the light of an inverted hammer and anvil, the hammer performing its work on the anvil of the superior jaw; and the machinery is perfect.

"But these parts, aided by the muscles connected with the chin, the tongue, the lips, and the fauces, have another duty of great delicacy and extent to perform, namely, that of articulation. This very essential function is the result of the combined action of all the muscles, though peculiarly delicate modifications, directed on the air that has been undulated into a sonorous state in its passage through the rima glottidis. In addition to this general view of the machinery and uses of the mouth in man, it will be necessary to examine, a little more minutely, the constituents of the skeleton of the mouth, and learn how that enduring portion in which the shape or ethnographic signs reside and become permanent is affected in different tribes, by the exercise of the functions exacted from the various parts. In this point of view, it may be useful to consider the mouth under three aspects: anterior, posterior and median. We shall, in that way, be better able to appreciate the peculiar mechanism displayed in its contrivance.

"Let us first inquire what are the different duties demanded of these parts, and then point out how their performance is provided for. 1. There are certain duties exacted by all mankind of the mouth, namely, seizing, dividing, and comminuting or grinding the food. For each of these actions there exists a central point of energy. The central point of energy for the act of seizing, resides in the median division, where the canine tooth is situated. That tooth has the most powerful single fang of any tooth in the whole dental range; and, from possessing a strongly pointed cusp, is peculiarly fitted for the act of transfixion. The canines are most conspicuously marked in some of the lower animals, and are known by the name of tusks. They are also powerfully marked in carnivorous animals, such as the dog, from which, indeed, they have obtained their appellation; but they are not less so amongst the feline and other carnivora. The canine tooth presents a marked feature in the countenance of all animals possessing it. Its position is beautifully adapted for seizing securely, without interfering with the vision of the animal, whilst he is grappling with his prey, it being placed aside, and not in the direct line of vision. This is a matter of great importance when these teeth require to be brought energetically into action, as the duties exacted of them are of primary importance, and must precede those of the others. On each side of this latero-median and essential tooth, are teeth which are of an intermediate character. The lateral incisor teeth, anterior to the canine, partake of a mixed character, of the canine and central incisor, and the small grinders or bicuspids, on the other side, are intermediate in character between the canine and the true molars or grinders; thus the canines at each corner pierce and transfix whatever is placed within their sphere of action, and hold it fast, while the anterior and intermediate accessories, the lateral incisors, divide it anteriorly, and the acute and compound cuspidated small grinders divide it posteriorly. The other two divisions of the dental range contain within each respectively a centre sphere of energy also, but very different in object. The anterior portion possesses the central incisors, but the power of their full action is not adapted to transfix, divide and tear, in a manner similar to that exercised by the powerful tooth we have just alluded to. They are the most distant from the power which acts on the jaw; and in the upper jaw they present a broad and chisel-shaped cusp, instead of the pointed and piercing cusp of the canine tooth, and the root even of the upper central incisor is about one-third less than that of the canine. On the whole, then, they have only about one-third of the power which the canine teeth have; and they are, consequently, only applicable to the division of small objects, which, as their name implies, is their true duty, assisted by the lateral incisors. The posterior division contains the machinery peculiarly adapted to the process of grinding or comminution. There is a central sphere of activity here likewise. That resides in the first large grinder, which is the standard tooth of division or comminution, crushing everything with great force upon which it is brought to act. In this duty it is materially and most efficiently assisted by the two small grinders in front, and the second and third large grinders behind. The centre of its action nearly corresponds with the centre of the great moving power of the jaw; so that there is a great concentration of force in this division. Such, then, are, generally, the duties exacted from these parts throughout all the races of mankind; and, having already explained the machinery by which the fulfilment of these duties is provided for, I come now to point out some peculiar considerations connected with the skeleton of the mouth, which will assist in explaining the ethnological signs exhibited in these parts.

"The most recognizable ethnological features are to be found in the anterior division, which presents, on the one hand, the prominent jaw and everted teeth of the negro, more particularly; and, on the other side, the crowded and irregularly-arranged teeth and perpendicular jaws of the Caucasian tribes. Both the other divisions of the dental arches, however, display, in like manner, characteristic features corresponding with these two states of existence, and which I shall endeavor successively to bring under attention. The anterior portions of both jaws may be considered as concentric arches. The arch formed by the edges of the teeth in the upper jaw being produced from a little longer radius than that formed by the edges of those in the under, it is evident that if these two arches are forcibly brought into approximation, the external arch of the superior jaw, with its contents, must yield outwardly; because, by forcibly applying the crown of an arch to the internal portion of another arch, you obviously afford to the internal arch an incontrollable mechanical advantage. It is also evident, that the forcible retention of any substance between these two arches must increase the intensity of the mechanical advantage, and the tendency of the lower to evert the upper. If we reflect on the peculiar anatomy of the parts, it will be seen, too, that the superior jaw yields to a much greater extent than the inferior. The median suture of the arch of the under jaw is soon consolidated; whereas, there remains a permanently ununited suture in the upper. But its plasticity is still further provided for, and in a more efficient way, by the presence of the intermaxillary bones, which, as their name implies, are situated in the centre between the bones of the true maxilla. These bones have not been generally recognized as separate existences in the adult human subject, though it is universally admitted that they are present in infants, and that they are occasionally to be found distinct throughout life. Some authors have even asserted that the absence of these bones form a characteristic in man. But they are of practical importance; and although, if carefully searched for, may be recognized throughout life, yet it is quite sufficient for my present purpose to know that they are recognized in early life, as that is the period at which the characteristic features are given to the osseous framework, and which continue to the end of our existence. Separate centres of ossification are to be met with here; and the radiations of these ossific growths are directed to the maxillary bones on each side, the median suture dividing them in the median line.

The transverse suture runs almost directly across the palate from the centre of the one alveolar process of the canine tooth to the other, comprehending, in that manner, the whole of the anterior region of the dental range, and implicating, in development, the centre of activity of the central region of the arch. The intermaxillary bones thus effect, by their presence, such a form of arrangement as to admit of great plasticity in the anterior arch of the mouth.

"I have already briefly adverted to the ordinary duties required of the teeth situated in the anterior portion of the mouth; and a moderate exercise of these may be considered their particular duties in a somewhat advanced stage of cultivated human existence. If these teeth are duly exercised, and proportionately with the others, we have then a development properly fitted for all social requisitions, at once affording the power of perfect articulation and perfect mastication. Articulation is entirely performed in this region of the mouth; and although mastication, properly so called, is not performed here, yet it is materially interfered with by any deviation from a regular arrangement even in this quarter. Thus, where there is an excess of luxury and indolence in social life, we find, from the want of functional exercise, that the jaws are not duly developed, and that early anchylosis of the different sutures is the result. From the osseous portion not being properly developed, space is not afforded for the accommodation of the second set of teeth. The second or permanent teeth, in the early stage of infantile existence, are arranged in the jaw behind the temporary teeth, and, consequently, in the arch of a circle of a shorter radius than that in which their predecessors are placed; and, being of larger dimensions, and confined within a smaller compass, they are forced to overlap each other in the very early and unextruded state. The indolence of the system will thus permit these teeth to creep into external existence in fetal arrangement, and they really do appear in that condition in the heads of a great proportion of the adults of civilized life. In such cases, unless corrected by art, the mouth approximates a carnivorous type, and inertness of comminution or grinding in the posterior or true masticating region is the consequence, from the impossibility of using the jaws in such an operation. Articulation, also, is sometimes materially interfered with from the unequal surface produced by the irregular arrangement of the anterior teeth upon which the tongue has to act. These irregularities of arrangement are some of the penalties of the irrational habits of social existence, and are never to be found amongst uncivilized races. Amongst races existing in a good climate, and where there is no deficiency of exercise or nourishment, a perfect development of the mouth, as well as of all other parts, follows. There is then a regular symmetrical arrangement of the teeth, the best adapted for perfect articulation, and for mastication, leaving the teeth perfectly arranged, and fully developed. The entire range then forms a perfect parabola, each tooth standing nearly perpendicularly to the portion of the alveolar ridge to which it is attached; and that, it is evident to me, is their normal development. That disposition is to be found in the vertical mouth of the Caucasian races, which, in my opinion, must have been the original development of mankind; and from which there is no difficulty in tracing all the varieties of the human species which have ever appeared on the face of the earth.

"Having attempted to explain the deviations from this type in the dental organs of social life by neglect of the exercise of the functions of the parts, I shall next endeavor to show how abuse, in a contrary line of habit, produces a development in an exactly opposite direction. I have described the arrangements which afford an extensive latitude of plasticity in the upper jaw, admitting of the parts to be modeled by the exercise of their ordinary functions. But in uncivilized life, extraordinary functions are called into action, and a great excess of energy is also thrown into those of an ordinary description.

"The ordinary duties required of the mouth in civilized life, as I have observed, are a moderate exercise of power for division, tearing, and comminution or grinding. In uncivilized life, however, there are superinduced upon these more powerful exactions, which have a great controlling influence over the development of the parts. Man, in the uncivilized state, has but few instruments or tools to assist him in operations of any kind, and his teeth are ready substitutes, which on all occasions, from infancy to old age, he most unscrupulously resorts to. He attacks the roughest materials of all kinds with his teeth. He uses them to form and to fashion those materials in all sorts of ways; and thus his mouth has a prehensile character. He also uses his teeth as instruments for punishing his enemies, seizing his prey, and separating the assimilative portions of his food from those which are not. In fact, they assist him on all occasions, and the forcible tearing which is habitually exacted from him, owing to his want of artificial instruments and the little assistance he derives from cooking, tends, most decidedly, to evert both the upper and the under jaw. Even at the earliest period of uncivilized existence, habits prevail which powerfully contribute to that extra development which produces the prominent mouth. We learn from actual observers, that the uncivilized mother suckles her offspring for the protracted period of two years or more, and that the prominent mouth does not exist in infancy; but its development is assisted by the habit of long sucking, which acts powerfully on the then very plastic condition of the bones of the jaw. Indeed, in social life, we have frequent examples of the modified effect of habits giving a like tendency in infancy to the protrusion of the anterior portion of the upper jaw, such as the child being allowed to suck its tongue or its fingers, or having to be fed for a long period from a hard bottle.

"Acts calculated to have an effect in moulding the jaw are not limited to infancy; they may extend throughout life; and the prominent development will always be found in proportion to the ratio of power of the under jaw; and we have not only seen how well the anatomical arrangement of the osseous parts admit of these mouldings, but we must be satisfied that the design is perfect in allowing of such modifications; otherwise they would have been constantly exposed to injury by force from without, and concussion from within. This plasticity, however, is limited. An examination of the skeletons of individuals with prominent jaws will demonstrate that it is a simple modeling of the original quantity of material which is effected. Beyond a full and perfect development of the parts, there is no peculiarity excepting the eversion of the material, or the placing of it in an altered position.

"To form the mouth of any other animal than man, difference of structure and a different specific quantity of material, become necessary. There is an accumulation of effect in particular directions, occasionally discoverable, which produces aberrations so extensive that they cannot be explained but upon the admission of the principle of hereditary transmission. Thus, in what I have stated, and in what may follow, it is not to be understood that the effects described as occurring are to be attributed entirely to the exercise of the functions of the parts during one generation, but as being the result of a succession. What the appreciable effect in one generation may be it is impossible to determine upon the data which we at present possess.

"If it be fully confirmed that the mouth of the infant negro is not prominent, it will be interesting to study the extent of the hereditary influence, and the period of development of that influence. I have hitherto alluded principally to the circumstances attending the development of the anterior portion of the mouth, including the incisors and canines; but characteristic habits of different races produce also corresponding deviations in its posterior region. A crowded state of the teeth, from want of due expansion and development of the bones in which they are implanted, producing an irregular pressure of one against another in the progress of growth; and a faulty organization of the dental tissues, increased by that irregularity, are amongst the effects of constitutional inactivity, depending on the habits of social life. But there is one serious evil which is only known in social life; and that is, the derangement interfering with the functions of the mouth, which is occasioned by the arrested development of the jaw, causing a deficiency of room for the development of the wisdom tooth. This, at times, causes great distress; and even death, by a slow process of torture. If that tooth at last struggle into external existence under such difficulties, it is, in a great majority of instances, found to be worthless, and only a source of torment to its possessor. On the other hand, however, we find that the rude uncivilized tribes of mankind possess a bold, well-developed, and healthy organization of structure in all the parts, and one free from irregular pressure. The wisdom tooth in them is so well developed, free in its position, and healthy in its structure, as to have induced some naturalists to consider themselves warranted in regarding it as a feature of approximation to the monkey tribe, although its good condition is nothing more than a feature of healthy development. The capabilities of this section of the mouth being limited simply to that of comminution, or grinding, it is not so much subject to the effects of abuse as the anterior portion of the dental range. Perhaps the only abuse of it is, that of exercise on food calculated to wear away the grinding surface of the teeth. The Hindu, the ancient Egyptian, and others, present examples of these surfaces being entirely worn away; and even of the teeth in the anterior and median portions of the mouth being reduced to truncated forms. The cause of this peculiar effect appears to be the roughness and grittiness of their food, and, in some cases, the almost exclusive consumption of food of a vegetable character. This is a powerful reason why man ought to be considered an omnivorous animal.

"Notwithstanding all I have said in favor of the more perfect development of the mouth in the rude and uncivilized tribes, they are, nevertheless, not altogether exempted from the ordinary diseases of the teeth. Independent of the habits I have referred to as affecting the arrangement of their teeth, and the development of their jaws, natural decay and disease occur, which we may refer to the state of health of the parents, the period of procreation, the circumstances under which their systems are at the time of production, and the inadequate nature of their nourishment, more especially in the early stages of existence. The general correctives of all these evils of development are energetic exercise, both of body and mind—residence in a healthful climate, pure air, and a due supply of wholesome and nourishing animal and vegetable food—not only in regard to individuals, but to successive generations. Combe remarks that 'no object can be presented to the philosophic mind more replete with interest than an inquiry into the causes of the differences of natural character.' Every one must feel the force of this remark, and not only assent to its truth, but also to the converse of the proposition, and agree that nothing can yield more pleasure to the philosophic mind than an inquiry into the effects which the natural character, with its accompanying habits and customs, produces on the human frame. I am persuaded that a study of the latter, conducted on proper principles, and elucidated by practical facts, would tend to the explanation of phenomena which have hitherto been referred to erroneous origin.

"The circumstances by which man is surrounded in uncivilized life do not afford opportunities for the cultivation and enjoyment of the higher faculties; and, accordingly, we find that a low retiring forehead is a concomitant of the prominent mouth. Another marked concomitant is that feature of countenance which is produced by the high cheek bones. The osseous frame-work of that prominence is composed of the portion of the superior maxillary bone in which the grinding teeth are implanted, and the true cheek bone or malar, which, with the zygomatic process of the temporal bone, forms the arch through which the temporal muscle or powerful levator of the under jaw passes. The first of these portions, namely, the portion of the superior maxillary bone, containing the molar teeth, is surmounted by the antrum or hollow ball of the cheek. The fangs of these molar teeth embrace the floor of this hollow, in the manner of beams or joistings. It is evident, that as these teeth are powerfully developed, the fangs will be strong and divergent, and thus increase the volume of the ball of the cheek. The exactions of uncivilized life produce that effect, and we, therefore, have this consequence. With the increase of this ball, we have a consequent protrusion of the bones which rest on this portion of the superior maxillary bone, namely, the malar, and through it the zygomatic process of the temporal. There is, however, a powerful concomitant increment to the protrusion of these latter bones, by means of the powerful action and development of the temporal muscle passing under it, and exercising its force with its consequent increase of bulk in expanding that arch.

"Although we have many well-authenticated cases recorded of these peculiar features of the human countenance being somewhat reclaimed or ameliorated by improvement in the circumstances of succeeding generations, yet there appears to be a greater or longer-continued tendency to the extra development of these than of any other. The prominent features in the high cheek bones of mountaineers are generally quite characteristic. The Scotch and Welsh highlanders of our own country are familiar examples. Exposure to a pure atmosphere produces in them keen appetites, which, by encouraging a vigorous mastication, may keep up the hereditary tendency. The concomitant of the flat nose with the prominent mouth, may be accounted for from the inversion of the superior portions of the intermaxillary bones forming the root of the nose; and this arises from the eversion of the inferior borders in which the teeth are placed. The bones thus, as it were, tilted, and receiving no permanent increase of material as they grow, equivalent to form new structures, are pressed upwards and backwards, and produce this derangement of feature by the inversion of the superior portions of the intermaxillary bones. The same causes will serve to explain the increased distance between the eyes of the uncivilized races, produced by the flattening and lateral expansion of the nasal bones; this being a necessary consequence of the expansion of all the other bones of the face.

"With regard to the other extreme of development which is generally to be observed in the mouths of civilized men, the concomitants are obvious, and quite as marked as those attending uncivilized men. It must accord with the experience of all, the precocity of intellect is very generally accompanied by an arrest of physical development and a languid constitution. When we meet with such an arrest of development and unhealthy secretion in the system generally, we must expect to find a similar arrest of development in the maxillary bones containing the cavities in which the teeth are lodged. This will occasion a deficiency of space for the proper arrangement and development of these organs, which, it is curious to remark, under all circumstances, follow the same train of growth as to size. They will also generally be found to be faulty in their structure when they arrive at maturity, or even as soon as they make their appearance externally.

"In addition to the ordinary diseases of the teeth called decay, the effeminacy of social life, the almost exclusive and unremitting exercise of the mental faculties, and a consequently superinduced morbid, nervous susceptibility, cause disease to appear in the sockets of the teeth, which produces their expulsion, although the bodies of the teeth themselves may be perfectly sound. That peculiarity of which both modern and ancient social life affords abundant examples, is frequently found to have existed in the sockets of the teeth of the ancient Egyptians,* but never to have been observed in races of men who have followed a natural course of life. I may remark here, that in the descendants of those who have lived long in social life, the cheek bones are not elevated, from the absence of encouragement to a powerful development of their basis; but the nose is elevated, owing to its root not being compressed as in the prominent mouth, and this feature is increased in its proportionate appearance from the absence of such a prominency.

"While that protrusion of the mouth is uncommon in civilized society, yet two varieties of malformation may occasionally be met with. The one caused by the projection of the upper jaw to a considerable extent over the under; and the other by that of the under beyond the upper. Generally speaking, both cases arise from an arrest of development in the jaw where expansion of the arch is deficient. The projecting upper jaw,

* Morton's Crania Americana.

however, as I have already stated, is very often the result of a habit of sucking the tongue or finger in infancy.

"It would be impossible, within my present limits, to appeal largely to history in support of all these facts and hypothetical enunciations; but if it were, I should hardly conceive it would be necessary, as a slight reflection must supply to the recollection of every one abundant general proofs in support of them, and which, on such an occasion as the present, is all that can be required. In conclusion, I cannot help simply remarking, having abstained for the sake of brevity from making many illustrative observations, as well as referential remarks upon the different points glanced at, that there is a curious train of results of peculiar forms of the mouth affecting the articulation of sounds, which it would be very interesting to study and to trace throughout all their modifications. The effects of the modifications are so very striking and decided, that I have no doubt an investigation into them would lead to many useful and interesting results."

### *Relation in respect of development between the cranium and the face.*

The relation in degree of development between the cranium and face offers some variety at different periods of life, and considerable diversity amongst animals; these differences mainly depending on the extent of development of the organs of smell and mastication. The comparative size of the cranium and face is also an indication of the relative proportion of the brain to the inferior organs of sense above referred to, and hence becomes a means of estimating the proportions subsisting between the mental and the animal faculties. The facial line of Camper, and the angle which it forms with the base of the cranium, are the most simple of the numerous means which have been adopted to determine this proportion. The ancients were fully aware of the importance of this measurement, and entertained the belief that an angle approaching to a right angle was an indication of a generous nature, and it was admitted to be a conspicuous mark of beauty. Indeed, in the statutes and models of their heroes and divinities, we find them exceeding natúre in this particular, and carrying their admiration of the principle beyond the precise limit of truth. Among the lower animals the facial angle of Camper is found to become more and more acute as we descend in the series of mammifera, and from the latter pass on to birds, reptiles, and fishes. But it must not be inferred that a mere elongation of the snout necessarily betokens deficient intellect, for this character simply indicates the existence of a highly developed organ of smell or mastication, without reflecting on the relative smallness of the brain. On the other hand, the great prominence of the anterior part of the cranium of the elephant, which gives him an aspect of superior intelligence, has no reference to the size of the brain, but to the unusual dimensions of the cells of the cranium ; an organization having relation to the huge size and great weight of the teeth, tusks, and trunk, which require large muscles to support and move them, and an extensive surface of attachment for their origin.

Bearing then in mind that the variation in the facial angle has reference to the development of the mouth and nose, as well as to that of the brain, it may be interesting to glance at the relative admeasurement of this angle in several of the races of man, and in the higher quadrumana. For example, the measurement of Camper's angle in the

| | |
|---|---|
| European is . . . . . . | 80° |
| Mogul . . . . . . | 75° |
| Negro . . . . . . . | 70° |
| European infant . . . . . | 90° |

The difference between the infant and adult European is plainly referrible to the deficient growth of the face in the former, and not to superiority in cerebral development. In illustration of this point I may remark that the angle in the

| | |
|---|---|
| Chimpanzee, adult, is . . . . | 35° |
| Chimpanzee, young . . . . . | 67° |
| Ouran Outan, (Pongo,) adult . . . | 40° |
| Ouran Outan, young, previously to the exchange of the deciduous for the permanent teeth . . . . . . . | 65° |

In many animals the facial angle is modified by the amount of expanson of the frontal sinus. This circumstance obviously affects its fidelity as a measurement of the development of the brain, and in like manner it is open to the objection of giving no idea of the breadth of the cerebral organ.—*Nasmyth's Researches on the Development, Structure, and Diseases of the Teeth.*

